# Real-World Spatial Synchronization of Event-CMOS Cameras through Deep Learning: A Novel CNN-DGCNN Approach

**DOI:** 10.3390/s24134050

**Published:** 2024-06-21

**Authors:** Dor Mizrahi, Ilan Laufer, Inon Zuckerman

**Affiliations:** 1 Department of Industrial Engineering and Management, Ariel University, Ariel 40700, Israel; ilanl@ariel.ac.il (I.L.); inonzu@ariel.ac.il (I.Z.); 2 Applied Physics Division, Soreq NRC, Yavne 81800, Israel

**Keywords:** dynamic graph convolutional neural networks (DGCNN), event-based sensing, sensor fusion, image alignment

## Abstract

This paper presents a new deep-learning architecture designed to enhance the spatial synchronization between CMOS and event cameras by harnessing their complementary characteristics. While CMOS cameras produce high-quality imagery, they struggle in rapidly changing environments—a limitation that event cameras overcome due to their superior temporal resolution and motion clarity. However, effective integration of these two technologies relies on achieving precise spatial alignment, a challenge unaddressed by current algorithms. Our architecture leverages a dynamic graph convolutional neural network (DGCNN) to process event data directly, improving synchronization accuracy. We found that synchronization precision strongly correlates with the spatial concentration and density of events, with denser distributions yielding better alignment results. Our empirical results demonstrate that areas with denser event clusters enhance calibration accuracy, with calibration errors increasing in more uniformly distributed event scenarios. This research pioneers scene-based synchronization between CMOS and event cameras, paving the way for advancements in mixed-modality visual systems. The implications are significant for applications requiring detailed visual and temporal information, setting new directions for the future of visual perception technologies.

## 1. Introduction

In the rapidly evolving field of imaging technology, integrating event-based cameras with traditional CMOS (complementary metal–oxide–semiconductor) sensors represents a significant research frontier with vast application potential. Traditional CMOS sensors have been the backbone of digital imaging for decades, providing high-quality, frame-based captures. However, they face challenges such as substantial power requirements, limited dynamic range, and susceptibility to motion blur in rapid-moving scenarios [1,2]. Conversely, event cameras, a relatively novel innovation, detect changes in light intensity at each pixel independently and asynchronously, only recording changes. This operation leads to lower power consumption, a much higher dynamic range, and the capability to capture fast-moving objects without blur [2].

Despite these advantages, previous studies on event cameras have encountered significant challenges. Traditional methods often aggregate events into grid-based representations for processing with standard vision pipelines, which can lead to inefficiencies and loss of information [3]. Additionally, there is a notable lack of a general framework to convert event streams into grid-based representations that can be learned end-to-end with task-specific networks [4]. Moreover, while event cameras have shown effectiveness for specific applications such as machine fault diagnosis and driving behavior characterization, these studies have highlighted difficulties in handling environmental noise and achieving high precision in real-world conditions [5].

The output from event cameras comprises a stream of “events” instead of conventional images. Each event represents a change in intensity at a pixel level, occurring independently of a frame structure. This output format can be challenging to integrate into many applications that traditionally rely on continuous frame-based video data, making it difficult to apply standard video processing techniques [3,6]. The potential of combining these two technologies lies in leveraging the continuous, high-resolution imagery of CMOS cameras with the high temporal resolution and motion sensitivity of event cameras. This hybrid approach could significantly enhance fields that require detailed, dynamic visual information, such as robotics [7,8,9]. While CMOS cameras provide essential high-resolution images for navigation and spotting obstacles, they can falter under rapidly changing light conditions or during high-speed situations. Event cameras, known for their quick response times and minimal delay, excel at capturing sudden changes in a scene, thus reducing motion blur.

Recent advancements in sensor fusion and visual-inertial odometry (VIO) have significantly enhanced the capabilities of autonomous vehicles. A notable approach integrates data from traditional CMOS cameras (frames) and event cameras (events) along with inertial measurements, creating a robust adaptive system. This system excels in complex lighting and high-speed conditions, significantly improving object detection and tracking accuracy. Central to this system is an 8-degrees-of-freedom (DOF) warping model that aligns the different data types by creating brightness increment patches, which help to minimize the differences between these outputs. This process allows for robust feature tracking by adaptively updating measurements based on the quality of tracked features, thus ensuring superior pose estimation accuracy compared to existing event-based VIO algorithms [10].

Nonetheless, achieving precise spatial synchronization between CMOS and event camera outputs remains a significant challenge. This difficulty primarily arises from the fundamental differences in their data acquisition modes. CMOS cameras capture uniform, time-synchronized frames, while event cameras record asynchronous, pixel-level changes triggered by variations in scene lighting. The asynchronous nature of event data does not align neatly with the synchronous frame rate of CMOS cameras, complicating real-time data fusion. Even with sophisticated models like the 8-DOF warping model, temporal alignment requires matching these sporadic, event-driven data with the uniform timestamps of frame data, necessitating complex interpolation and prediction methods that are yet to be perfected. These challenges necessitate advanced synchronization techniques beyond traditional calibration methods, which often fall short in dynamic, real-world conditions where both types of cameras must operate in unison [11]. Previous attempts to transform event camera data into pseudo-images for applying conventional calibration techniques have proven complex and often imprecise, particularly in handling real-time, dynamically changing environments [11,12].

Our work aims to bridge this gap by directly synchronizing the output of event cameras with that of CMOS cameras, bypassing the intermediate transformation steps. We propose a direct method for aligning these disparate data streams, enhancing the accuracy and efficiency of the process. Our approach utilizes deep learning models to interpret and synchronize the raw outputs from both camera types, focusing on real-time, real-world applicability [4,13]. We introduce innovative neural network architectures that are specifically designed to handle the unique characteristics of each camera’s output, facilitating a seamless integration of these technologies. Additionally, the integration of dynamic vision sensors into unmanned aerial vehicles optimizes the estimation of dynamic interactions in real time, demonstrating the effectiveness of event cameras in scenarios that require rapid and precise adjustments [3,14].

Our findings show that this direct synchronization method significantly improves the accuracy and efficiency of data integration from both camera types, overcoming the limitations of previous approaches. The proposed deep learning architectures enable end-to-end learning and adaptive processing, leading to enhanced feature extraction and event interpretation. Experimental results have demonstrated high fault diagnosis accuracies in machinery monitoring, comparable to traditional accelerometer data, while also being effective in driving behavior characterization and autonomous navigation tasks. These advancements suggest that the method can be applied in diverse real-world scenarios, potentially offering reliable performance in dynamic environments [5,13].

## 2. Materials and Methods

### 2.1. Workflow Overview

**Figure 1:**
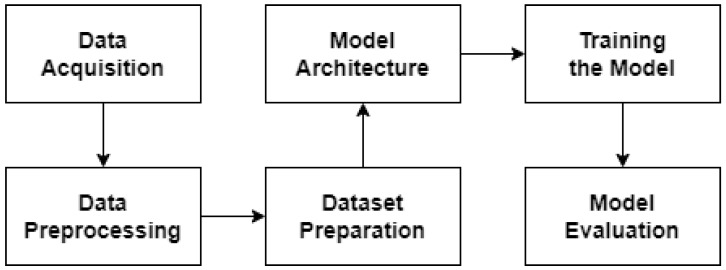
Flowchart—workflow overview.

The flowchart (see Figure 1) outlines the key steps involved in the spatial synchronization of CMOS and event cameras using the CNN-DGCNN model. This workflow covers the entire process from data acquisition to model evaluation.

The process begins with data acquisition, where we collect high-resolution video data from CMOS cameras (480 p and 1080 p) and corresponding event data from event cameras. Next, during the data preprocessing step, we segment CMOS video frames into 100 × 100 windows and label these segments with precise shifts. Following this, in the dataset preparation phase, we create training and testing datasets from the segmented and labeled data, with the training set containing 3 million observations and the testing set comprising 600,000 observations.

In the model architecture stage, we define a convolutional neural network (CNN) to extract features from CMOS data and a dynamic graph convolutional neural network (DGCNN) to process event data. These outputs are then integrated using a multi-layer perceptron (MLP). During the model training phase, we initialize the model parameters and train the model using the Adam optimizer, applying early stopping and a learning rate scheduler to enhance performance and prevent overfitting. Finally, in the model evaluation step, we assess the model’s performance on the test set by computing the calibration error and analyzing the results to ensure accuracy and reliability.

### 2.2. Event Camera Data Format

Event cameras are specialized sensors that capture local variations in luminance over time. Differing from conventional cameras, which use shutters to take pictures, event cameras consist of pixels that function autonomously and asynchronously. These pixels activate only in response to changes in light intensity, otherwise remaining passive. This mechanism allows for a highly efficient and sparse representation of video sequences which is particularly suited to natural scenes. In essence, when any pixel detects a variation in brightness, it generates an event. This information is aggregated as a sequence of events:

(1) $${\left\{e_{i}\right\}}_{i=1}^{N}={\left\{{(x}_{i},y_{i},t_{i},p_{i})\right\}}_{i=1}^{N}$$

In Equation (1), ${(x}_{i},y_{i})$ represents the spatial coordinates of the event, $t_{i}$ denotes the event’s timing (with microsecond precision), and $p_{i}$ signifies the intensity change at the pixel. Consequently, the output from an event camera is a streamlined series of events, structured as follows, with the objective being to apply vision-based downstream processing to these data, specifically for 2D regression tasks.

### 2.3. Dataset Creation

To facilitate the training of our proposed models, a comprehensive and accurately labeled dataset was essential. This dataset needed to encapsulate both images from the CMOS camera and the corresponding events captured by the event camera, alongside the precise movements on each axis to ensure effective spatial synchronization.

#### 2.3.1. Data Sources

**Figure 2:**
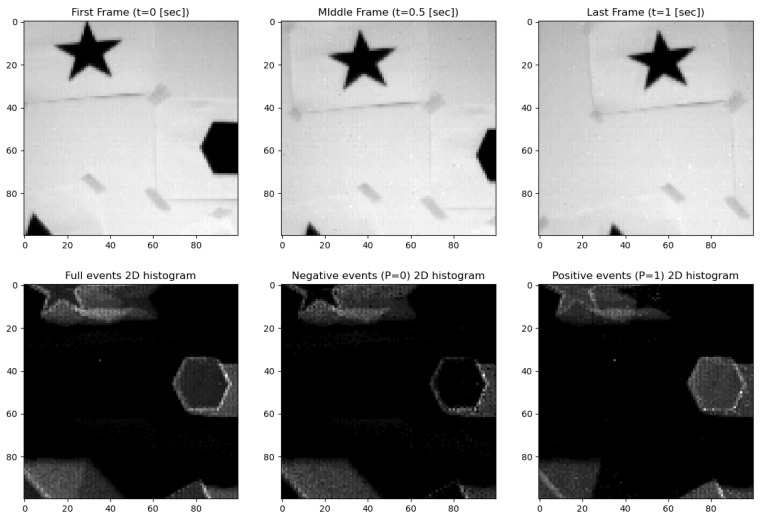
Video frame and corresponding 2D event histogram maps (“shapes_rotation”).

**Figure 3:**
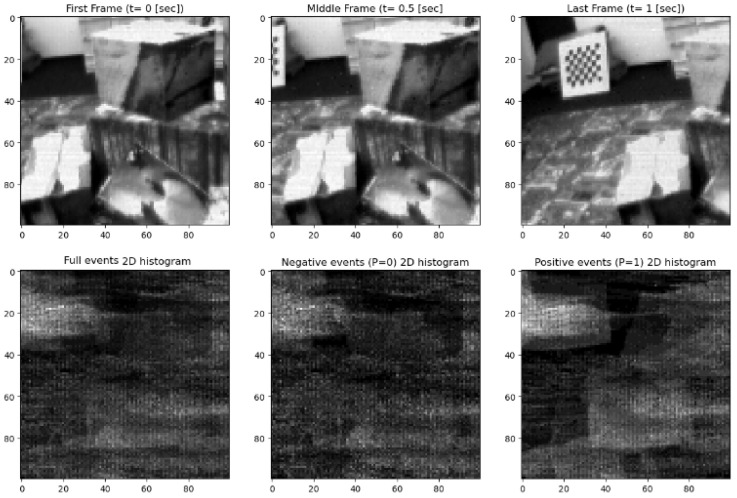
Video frame and corresponding 2D event histogram maps (“boxes_rotation”).

For our study, we selected three datasets from the DAVIS 240C Dataset [12,13], valued for its unique integration of a conventional CMOS camera with an event-based sensor. This integration offers dual perspectives on visual data capture, as demonstrated in Figure 2 and Figure 3.

The “shapes rotation” dataset [15], visualized in Figure 2, presents simple geometric shapes against a wall. This dataset provided a controlled environment in which to test the basic synchronization capabilities of our models. Figure 2 illustrates this setup with three distinct frames: the first frame (t = 0 s) shows a single stationary shape; the middle frame (t = 0.5 s) captures the shape mid-rotation; and the last frame (t = 1 s) depicts the rotation’s conclusion, with a change in orientation of the shapes. Below the video frames, corresponding 2D event histogram maps split into full events, negative events (P = 0), and positive events (P = 1). These histograms capture the pixel-wise changes in intensity due to movement, with the negative and positive events representing decreases a d increases in intensity, respectively.

“Boxes rotation” [15,16], as seen in Figure 3, is set within a complex, texture-rich environment to provide a multifaceted pattern of movements, challenging our models under diverse textural conditions. In Figure 2, the first frame (t = 0 s) shows the initial static scene. The middle frame (t = 0.5 s) captures slight movement, primarily due to the camera’s motion. The last frame (t = 1 s) includes more noticeable movement, again primarily from the camera’s perspective. This series of frames illustrates the scene as observed by the camera over a one-second interval. The corresponding 2D event histogram maps below each frame represent the full events, negative events (P = 0), and positive events (P = 1), highlighting the pixel-wise changes in intensity due to the movement. To produce a tagged dataset with precisely labeled shifts, we reduced and cropped the CMOS segments to smaller windows of 100 × 100 pixels. This reduction was necessary to align the CMOS data accurately with the event camera data and to manage computational resources effectively. Figure 2 and Figure 3 reflect these 100 × 100 segments from higher-resolution movies, providing a proof of concept within the study’s scope. Thus, this context also applies to Figure 3.

The “outdoors walking” dataset records the conditions of a sunny urban environment, including changes in natural light and the active nature of outdoor scenes. Although this dataset is not shown here (refer to Figure 2 and Figure 3 for impressions of similar datasets), it adds value to the previous two by offering a real-world setting with uncontrolled lighting and movement. This helps us to test the effectiveness of our synchronization methods under less predictable conditions. These datasets encompass a broad spectrum of visual scenarios, from the simplicity and control of rotating shapes to the complexity of textured motion and the unpredictability of outdoor settings. This strategic curation of datasets was pivotal in developing our synchronization approaches, ensuring that they are effective and resilient in various real-life applications. The datasets provide a solid foundation for training and evaluating our models, confirming their effectiveness for practical use.

#### 2.3.2. Data Segmentation

We segmented the video data into one-second intervals, each comprising 22 frames, adhering to the dataset’s frame rate of 22 FPS. Given the original frame dimensions of 240 × 180 pixels, we further processed these frames into sub-frames or windows of 100 × 100 pixels. This segmentation was achieved by sliding the window across the frame in 20-pixel increments, ensuring thorough coverage and variety in the captured data.

#### 2.3.3. Labeling and Variations

**Figure 4:**
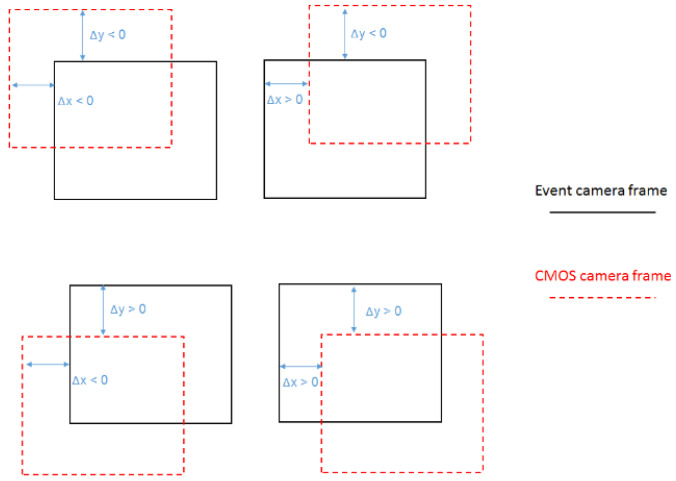
Illustration of frame shifting to produce labeled data, showing the positions of the CMOS camera frame (dotted line) relative to the event camera frame (solid line) for a diverse set of shifts along the X and Y axes.

In our dataset, each window extracted from the video data underwent a systematic frame shifting process relative to the event image. This process, as elucidated in Figure 4, involved shifting the CMOS camera frame with respect to the event camera frame across a defined range along both the X and Y axes. Figure 4 provides a visual representation of the four primary shift scenarios implemented to produce our labeled dataset. Each illustration demonstrates a CMOS camera frame (dotted line) and an event camera frame (solid line) with specific shifts along the X and Y axes:

1. Top left illustration: Here, the CMOS camera frame is shifted to the left (Δx < 0) and upwards (Δy < 0) relative to the event camera frame, depicting a negative shift along both axes.
2. Top right illustration: The CMOS camera frame is shifted to the right (Δx > 0) and upwards (Δy < 0) with respect to the event camera frame, illustrating a positive shift along the X axis and a negative shift along the Y axis.
3. Bottom left illustration: This scenario shows the CMOS camera frame shifted to the left (Δx < 0) and downwards (Δy > 0) in comparison to the event camera frame, indicating a negative shift along the X axis and a positive shift along the Y axis.
4. Bottom right illustration: The CMOS camera frame is shifted to the right (Δx > 0) and downwards (Δy > 0) relative to the event camera frame, representing a positive shift along both the X and Y axes.

For each 100 × 100 window, the frame shifts range from 0 to 70 pixels in any direction, resulting in an extensive range of 140 × 140 unique shift variations, spanning from −70 to +70 pixels for each axis. This systematic shifting creates a precisely labeled dataset that documents each window with known positional shifts. These labeled samples are instrumental for training spatial synchronization models that accurately align CMOS and event camera data.

Each depicted frame shift represents a specific labeled instance in our dataset, capturing a diverse array of relative positions between the CMOS and event camera frames. Collectively, these labeled instances encompass a full spectrum of possible frame alignments within the stipulated range. This structured labeling approach ensures that our dataset supports the training of a generalized model capable of handling a wide range of real-world synchronization scenarios.

By leveraging the entire collection of films available in the DAVIS240C dataset, we assembled a dataset intended to support the training of a model that generalizes across different visual contexts. This approach aims to enhance the performance and applicability of our synchronization methodology by incorporating a diverse range of visual scenarios.

To evaluate the proposed method, we created a large dataset for both training and testing. Approximately 3 million observations with various movements were selected for the training set, ensuring an equal distribution from all three movies (“shapes rotation,” “boxes rotation,” and “outdoors walking”). This balanced selection helps the model to generalize across different visual scenarios. For the testing phase, we used 600,000 observations, also evenly distributed among the three movies. This dataset allowed us to evaluate the performance of our CNN-DGCNN method across different and dynamic environments.

### 2.4. Network Architecture for CMOS and Event Camera Calibration

The network architecture detailed in this manuscript features a dual-input system designed specifically for the task of calibrating data from two distinct camera technologies: the conventional CMOS (complementary metal–oxide–semiconductor) camera and the innovative event camera. This setup is essential for effectively processing and integrating the disparate data types that these cameras capture. Figure 4 displays the DGCNN-CNN calibration network architecture, which employs a dual-input strategy to process and synchronize inputs from both CMOS and event cameras efficiently. The diagram details the data flow within the network, showing separate pathways for CMOS video and event camera data that converge to a unified calibration output. This visual representation elucidates the system’s architecture, highlighting how distinct data streams are integrated and processed.

The dynamic graph convolutional neural network (DGCNN) processes event camera data by updating the graph structure as new data are received. This method identifies spatial relationships within the event data, which is necessary for accurate calibration. The DGCNN builds a graph G=(V,E), where V represents the feature vectors of the vertices (events) at layer l and E represents the edges (connections between events). Each event detected by the camera is represented as a vertex in V, and the connections between these events are the edges in E.

The initial feature vector ${h_{i}}^{o}$ for each vertex i is based on the raw event data, including spatial coordinates $\left(x_{i},y_{i}\right)$, the timestamp ${(t}_{i})$, and the intensity change ${(p}_{i}$). The EdgeConv operation, which updates the feature vectors of the vertices, is defined as:

(2) $${h_{i}}^{l+1}={max}_{j:\left(i,j\right)\in E}ReLU\left({h_{i}}^{l}+\emptyset \left({h_{j}}^{l}-{h_{i}}^{l}\right)\right)$$

Here, ${h_{i}}^{l}$ is the feature vector of vertex i at layer l; ${h_{j}}^{l}$ is the feature vector of a neighboring vertex j; and ∅ is a neural network function, often a multi-layer perceptron, that computes the edge features based on the difference between the feature vectors of neighboring vertices. ReLU (Rectified Linear Unit) is an activation function that introduces non-linearity into the model, allowing it to learn more complex patterns. The max operation aggregates features from neighboring vertices, helping the network understand spatial relationships effectively.

Within each DGCNN layer, the feature vectors of the vertices are updated by considering the features of their neighboring vertices. For each vertex i, a new feature embedding ${e_{i}}^{l+1}$ is computed as:

(3) $${e_{i}}^{l+1}={max}_{j:\left(i,j\right)\in E}\emptyset \left({{{h_{i}}^{l},h}_{j}}^{l}-{h_{i}}^{l}\right)$$

This step involves computing the new feature embedding for each vertex by looking at the differences in the feature vectors of neighboring vertices and applying the function ∅. After processing through all the DGCNN layers, the final feature vector for each vertex is obtained. The final feature vector ${h_{i}}^{L}$ for each vertex i is represented as:

(4) $${h_{i}}^{L}=AGGREGATE(\left\{{e_{i}}^{l}\right|l=1,\ldots ,L\})$$

The AGGREGATE function combines the embeddings from all layers l to form the final feature vector ${h_{i}}^{L}$ for each vertex i. On the left side of the illustration presented in Figure 4, the input from a CMOS camera is depicted as a 1 s video clip segmented into 22 frames, each with a resolution of 100 × 100 pixels. These video data undergo processing through multiple CNN blocks designed to progressively refine the data. Initially starting with 64 channels, the architecture expands to 256 channels, all using a 3 × 3 kernel size. Each block is engineered to enhance the network’s understanding of the video by extracting features at progressively higher levels of abstraction. As the network advances, the depth of the feature maps increases, enabling the capture of increasingly complex patterns and details. This refinement process, which includes convolution, batch normalization, and max-pooling, effectively reduces dimensionality and accentuates key features.

On the right side, the input from the event camera is represented as a point cloud with n-by-3 dimensions. This sparse and asynchronous event data undergo processing through a series of EdgeConv layers, which are structured to progressively increase in complexity from 64 to 512 output channels. The EdgeConv layers are tasked with constructing and updating a dynamic graph that captures spatial relationships within the event data, enabling the network to adaptively learn and integrate event information over time. This is vital for accurate temporal tracking of events captured by the event camera.

The final layer of the DGCNN outputs features that are fused with the CNN-processed CMOS data through a multi-layer perceptron (MLP) to predict the spatial calibration parameters (∆x,∆y):

(5) $$\left(∆x,∆y\right)=MLP\left(h_{cmos},h_{DGCNN}\right)$$

**Figure 5:**
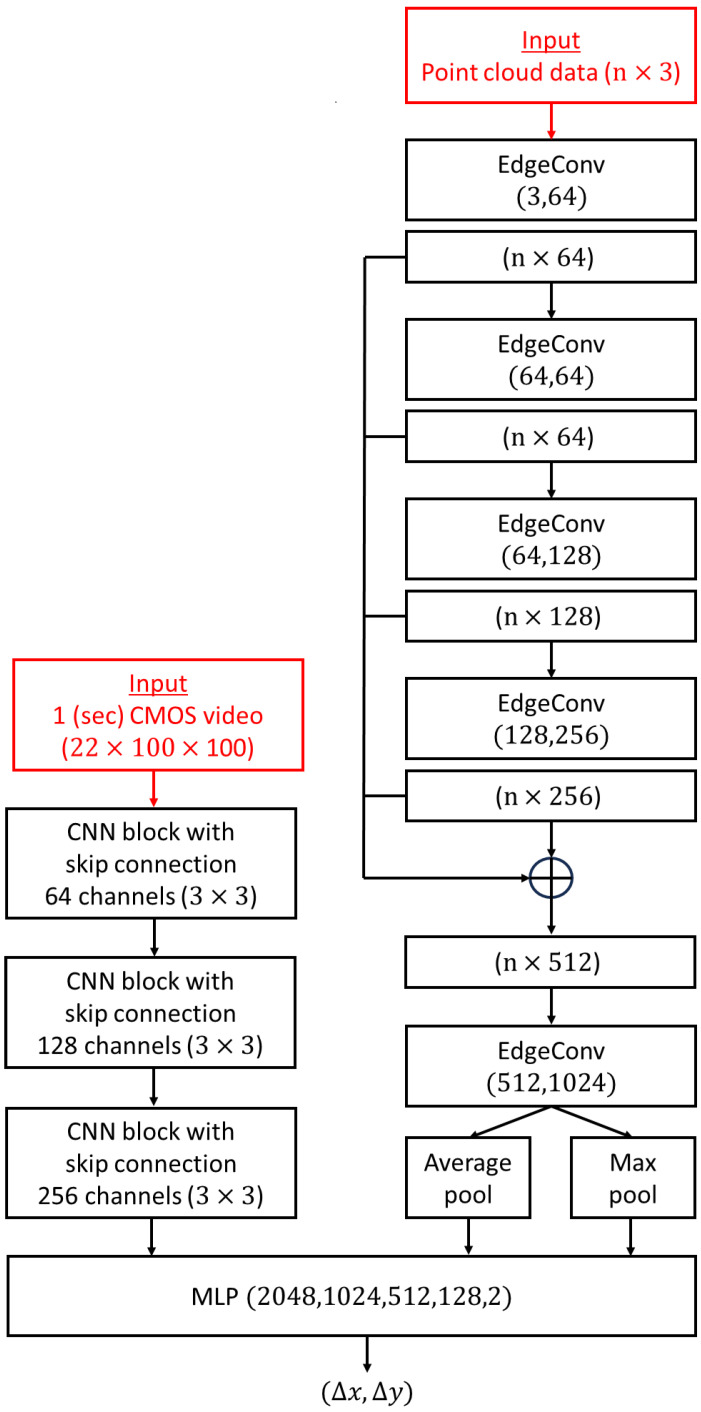
DGCNN-CNN calibration network architecture.

Here, $h_{cmos}$ and $h_{DGCNN}$ represent the feature vectors from the CNN and DGCNN, respectively. This fusion (as illustrated in Figure 5) enables precise alignment of CMOS and event camera data, ensuring accurate spatial synchronization.

#### 2.4.1. Convolutional Neural Network (CNN) for CMOS Data Processing

The CMOS camera data are processed through a series of convolutional neural network (CNN) blocks, each designed to handle 1 s video segments consisting of 22 frames at a resolution of 100 × 100 pixels. These blocks include convolutional layers with a 3 × 3 kernel, followed by batch normalization, which ensures consistent learning by centering and scaling the layer inputs [17,18]. Batch normalization helps to maintain a steady learning process across the network. After normalization, a max-pooling operation with a 2 × 2 kernel reduces the spatial dimensions of the feature maps, decreasing computational demands and reducing the risk of overfitting.

**Figure 6:**
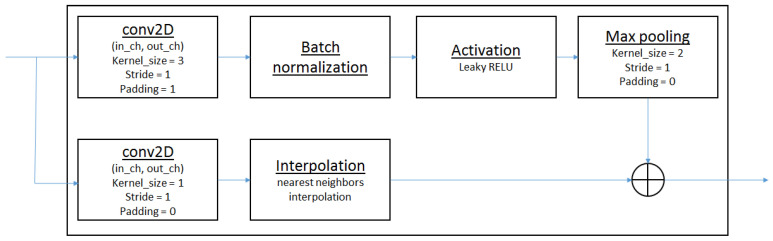
Visualization of a single CNN block with skip connection.

Each CNN block incorporates a skip connection, as shown in Figure 6. The skip connection provides a direct gradient path, bypassing one or more layers, which prevents vanishing gradient issues in deeper networks. To ensure compatibility with the main CNN output, an additional 2D convolutional layer with a 1 × 1 kernel adjusts the feature map dimensions within the skip pathway. This element-wise addition merges the bypassed information with the main pathway’s output, maintaining data integrity while benefiting from the learned features.

Figure 6 illustrates a single CNN block with a skip connection, detailing its structure. The input initially passes through a 2D convolutional layer, capturing basic spatial features. Batch normalization stabilizes the learning process, and an activation function introduces non-linearity. A max-pooling operation follows to reduce the feature map dimensions and data volume for subsequent layers. The skip pathway contains a 2D convolutional layer to adjust feature map dimensions, aligning them with the main pathway output. This ensures that both immediate and deeper features contribute to the final representation of the video data, encapsulating low-level details and high-level abstractions. By combining these components, the network architecture is optimized for processing CMOS camera data, facilitating accurate calibration when integrated with event camera data.

#### 2.4.2. Dynamic Graph CNN (DGCNN) for Event Data Processing

Event camera data differ significantly from traditional camera data due to their unique characteristics. Represented as a point cloud (where each point has dimensions n-by-3), the data are processed using a specialized neural network called a dynamic graph CNN (DGCNN). Unlike standard CNNs, the DGCNN is well-suited for handling the unstructured nature of point cloud data. Inspired by successful architectures used for gesture recognition with event cameras [19], the DGCNN is adept at capturing spatial relationships and patterns within irregular and sparse data distributions [20]. It constructs a graph that dynamically adjusts as new data are received, allowing the network to remain current with the most recent spatial relationships between points. The network architecture gradually increases in complexity, starting with layers configured for simpler feature detection (3 × 64) and then progressing to layers capable of identifying more intricate features (512 × 1024). This step-by-step increase in layer complexity enables the DGCNN to analyze and understand the full depth of event data comprehensively.

#### 2.4.3. Network Fusion and Calibration Output

After extracting features from the standard video using the CNN blocks and from the event data using the DGCNN, these features are combined for calibration in a multi-layer perceptron (MLP). The MLP in our system consists of layers with 2048, 1024, 512, 128, and 2 neurons. This structure processes data from both sources and outputs spatial calibration parameters (Δx, Δy). This step aligns spatial information from the CMOS camera with temporal data from the event camera, creating a detailed representation of motion and space. By merging these data streams, our network supports real-time spatial calibration, potentially enhancing systems that rely on mixed-modality visual information, such as autonomous navigation, robotics, and augmented reality.

### 2.5. Computational Setup and Training Parameters

To evaluate our CNN-DGCNN model, we used a local machine equipped with a 13th Gen Intel Core i9-13900K processor, 128 GB of RAM, and an Nvidia GeForce RTX 4080 GPU with 16 GB of RAM. These specifications ensured efficient handling of large datasets and complex computations.

Training was conducted using PyTorch version 1.10. We set the number of epochs to 100 and used early stopping based on validation loss to prevent overfitting. The Adam optimizer was chosen for its efficiency in managing large datasets and its adaptive learning rate capabilities, set at an initial rate of 0.001. We used a batch size of 64 to balance memory usage and computational efficiency. Early stopping was implemented with a patience of 10 epochs, meaning training would halt if there was no improvement in validation loss over 10 consecutive epochs. Additionally, a learning rate scheduler, ReduceLROnPlateau, was applied with a factor of 0.1 and patience of 5 epochs to dynamically adjust the learning rate and refine the training process.

During training, we closely monitored the model’s performance using validation loss. Specifically, we tracked the difference between the predicted and actual calibration parameters (Δx, Δy). This allowed us to apply early stopping when there was no significant reduction in validation loss, ensuring that the model did not overfit to the training data. By dynamically adjusting the learning rate based on validation performance, we were able to fine-tune the model for optimal accuracy and efficiency.

## 3. Results

Our study investigates how the spatial distribution of events within an image, described through entropy, impacts the precision of video calibration algorithms. Notably, areas with denser event clusters, which correspond to lower entropy values, improve the calibration’s accuracy. This empirical observation is supported by the entropy equation detailed in Equation (2), which calculates the entropy based on the distribution of events:

(6) $$Entropy=-\sum_{x=1}^{m}\sum_{y=1}^{n}p\left(x,y\right)*{log}_{2}\left(p\left(x,y\right)\right)$$

where:

m is the number of rows in the input video and the corresponding two-dimensional histogram $p\left(x,y\right)$. In this case, m is defined as 100.
n is is the number of columns in the input video and the corresponding two-dimensional histogram $p\left(x,y\right)$. In this case, n is defined as 100.
$p\left(x,y\right)$ is the ratio of the number of events at point (x,y) in the two-dimensional histogram to the total of all events in the histogram.

To evaluate the calibration process precisely, we measure the calibration error using the following distance formula:

(7) $$\delta_{cal}=\sqrt{{\left(∆x-\tilde{∆x}\right)}^{2}+{\left(∆y-\tilde{∆y}\right)}^{2}}$$

where:

$\delta_{cal}$ represents the calibration error distance;
∆x,∆y are the labeled calibration shift values per each window;
$\tilde{∆x},\tilde{∆y}$ are the estimated calibration shift values per each window.

**Figure 7:**
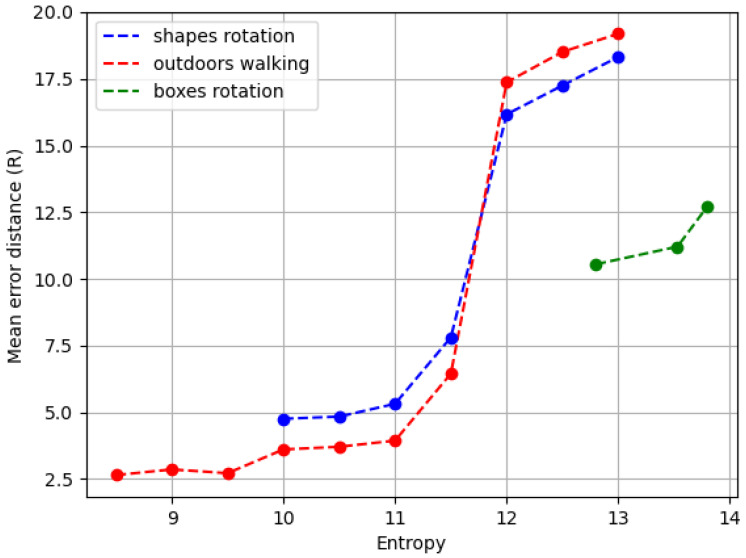
Calibration results as a function of entropy value and dataset.

The effect of entropy on calibration precision is captured in Figure 7, which portrays a significant finding from our study: the calibration error distance increases with entropy, yet this relationship exhibits variability depending on the dataset. Thus, this graph serves as an indicator of how different environmental conditions can uniquely impact calibration performance.

In “shapes rotation” (represented by the blue dash–dot line), the calibration error remains relatively stable and then shows a gradual increase as the entropy grows. The “outdoors walking” dataset (indicated by the red dash–dot line) starts with a lower error distance at low entropy levels, but as entropy increases, the error distance rises more sharply than in “shapes rotation”. This sharp increase suggests that the model has more difficulty achieving precise calibration when events are uniformly distributed, which is common in outdoor environments.

Most notably, the “boxes rotation” dataset (marked by the green dash–dot line) shows a distinct pattern. The calibration error starts off minimal and increases at a slower rate than the other datasets, despite having the highest entropy. However, once reaching a certain threshold, there is a noticeable jump in the error distance, after which it stabilizes. This unique trend may be attributed to the specific characteristics of the dataset, such as the presence of high-contrast textures, which could initially assist in the calibration process but become less beneficial as entropy increases beyond a certain point.

These variations in error response to entropy across the datasets emphasize the complexity of calibration tasks. Different scene characteristics, such as the presence of distinct textures in “boxes rotation” or the more dynamic elements in “outdoors walking”, are likely influencing factors in how well the calibration algorithm can perform.

Table 1 complements the graphical data, summarizing the average and variability of entropy and event counts for the datasets. “Shapes rotation” records a mean entropy of 12.69 (SD: 0.51), with an event count average of 95K (SD: 37K). “Outdoors walking” shows a lower average entropy of 12.11 (SD: 0.92) and a higher average event count of 145K (SD: 67K). “Boxes rotation” stands out, with the highest average entropy of 13.23 (SD: 0.05) and the largest average number of events at 712K (SD: 334K).

When comparing the data from Table 1 with the calibration error trends depicted in Figure 7, we can see a clear connection between entropy and calibration accuracy. This observation reinforces the importance of considering the distribution of events when designing and refining calibration methods for video analytics.

## 4. Discussion

Our study reveals that the complex interaction between entropy, event density, and calibration accuracy significantly impacts the outcomes of video data processing. Regions within a video frame with dense event distributions provide strong reference points, enhancing calibration accuracy. This is particularly evident when event cameras capture dense clusters of changes, which serve as precise anchor points for the calibration process. These “high information” zones not only increase the overall entropy, but also directly enhance the calibration effectiveness.

The effectiveness of dynamic graph CNNs (DGCNNs) in handling event data has improved real-time spatial synchronization and fostered the development of algorithms that adaptively respond to variations in event density and distribution, enhancing synchronization accuracy across diverse environments [3,21]. Our empirical tests confirm that calibration quality is heavily dependent on both the volume and spatial concentration of events, with denser distributions yielding better outcomes. These insights underscore the value of integrating CMOS and event camera technologies for applications demanding high precision and responsiveness, and they pave the way for future research into optimizing mixed-modality visual systems [3,21].

These calibration advancements extend into environmental monitoring, where accurate, real-time calibration of sensor data across various modalities is critical. For instance, edge-detection algorithms calibrated for specific underwater conditions have enabled more precise monitoring of coral ecosystems, contributing to conservation efforts [22]. Similarly, precise calibration in atmospheric monitoring using techniques like Fabry–Perot interferometers, which measure light wavelengths to detect CO_2_ levels, plays a vital role in efforts to mitigate the greenhouse effect [23].

Furthermore, the scope of these advancements has broad implications for industries such as autonomous vehicle navigation, robotic surgery, and augmented reality, where enhanced calibration techniques enable more precise and real-time interactions between event and CMOS cameras [24,25]. The integration of these technologies with emerging IoT devices and smart city infrastructure has also opened up transformative opportunities for urban development. Enhanced calibration techniques are essential for effective traffic management and interactive public safety solutions, ensuring accurate, real-time data processing. Techniques like the attention-based encoder–decoder network using atrous convolution are being utilized to effectively calibrate data from urban environments, aiding in urban water management [26].

In the Internet of Vehicles (IoV), innovative traffic management solutions, such as the dynamic ant colony optimization algorithm with a look-ahead mechanism, are revolutionizing urban traffic management by leveraging real-time data from wireless sensor networks. This enhances traffic signal control efficiency and reduces carbon emissions, highlighting the essential role of advanced calibration in maintaining data accuracy and reliability in these systems [27].

However, our study has limitations due to its reliance on specific datasets and conditions. Future research should extend these findings across a wider range of scenarios, including those with challenging lighting and fast-moving subjects. Additionally, improving the computational efficiency of our proposed architecture will be important to ensure its utility in real-world, time-sensitive applications [28].

Looking ahead, we suggest exploring different deep learning architectures to improve the calibration speed and efficiency [28]. Addressing non-linear distortions from camera lenses is also crucial [29,30]. Testing these improvements in practical settings will help to determine their real-world effectiveness [31,32]. Integrating this technology into wearable devices could expand its uses. Wearables with advanced calibration capabilities could enhance user experiences in fitness tracking, health monitoring for prosthetic adjustments, and interactive environments, blending digital and physical interactions [33].

In conclusion, our research on improving calibration methods is key to advancing 3-D reconstruction capabilities, as demonstrated by our study and supported by related literature on photometric stereo [34]. These improvements demonstrate the reliability of our methods and provide greater insight into how light interacts with surfaces, thereby enhancing 3-D imaging techniques. In future research, it will be important to refine visual systems in order to better adapt to the complexities of modern environments.

## Figures and Tables

**Table 1:** Mean and SD of entropy and events count by dataset.

Dataset/Metric	Mean Entropy	STD Entropy	Mean Number of Events	STD Number of Events
**Shapes rotation**	12.69	0.51	95K	37K
**Outdoors walking**	12.11	0.92	145K	67K
**Boxes rotation**	13.23	0.05	712K	334K

## Data Availability

The raw data supporting the conclusions of this article will be made available by the authors on request.
